# *PLTP* Variants rs6065904 and rs378114 Are Associated with Acute Coronary Syndrome Risk and Altered Glucose and Triglyceride Levels in a Mexican Population

**DOI:** 10.3390/biomedicines14092025

**Published:** 2026-09-09

**Authors:** Gilberto Vargas-Alarcón, Oscar Pérez-Méndez, Rosalinda Posadas-Sánchez, Julio Cesar Domínguez-Méndez, Marva Arellano-González, Galileo Escobedo, Teresa Juárez-Cedillo, José Manuel Fragoso

**Affiliations:** 1 Research Direction, Instituto Nacional de Cardiología Ignacio Chávez, Mexico City 14080, Mexico; gvargas63@yahoo.com; 2 Department of Molecular Biology, Instituto Nacional de Cardiología Ignacio Chávez, Juan Badiano No. 1, Tlalpan, Mexico City 14080, Mexico; opmendez@yahoo.com (O.P.-M.); dominguezmendezjc@gmail.com (J.C.D.-M.); marva_01@hotmail.com (M.A.-G.); 3 Department of Endocrinology, Instituto Nacional de Cardiología Ignacio Chávez, Mexico City 14080, Mexico; rossy_posadas_s@yahoo.it; 4 Laboratory of Immunometabolism, Research Division, Hospital General de Mexico, Dr. Eduardo Liceaga, Mexico City 06720, Mexico; gescobedog@msn.com; 5 Research Unit in Epidemiology and Health Services, Area of Aging, Centro Médico Nacional Siglo XXI, Instituto Mexicano del Seguro Social, Mexico City 06720, Mexico; terezillo@yahoo.com.mx

**Keywords:** acute coronary syndrome, phospholipid transfer protein, genetic susceptibility

## Abstract

**Background**: Phospholipid transfer protein (PLTP) facilitates lipid transfer among plasma lipoproteins, but the contribution of this protein to coronary heart disease (CHD) remains controversial. In this context, evaluating single nucleotide polymorphisms (SNPs) in the *PLTP* gene, specifically rs6065904 *A/G* and rs378114 *T/C*, may hold clinical utility for predicting CHD risk. Nevertheless, currently evidence on the association between these variants and cardiovascular outcomes remains scarce. To address this gap, we investigated whether the rs6065904 *A/G* and rs378114 *T/C* SNPs of the *PLTP* gene are associated with the incidence of acute coronary syndrome (ACS) and explored whether this potential relationship is mediated by plasma lipid levels. **Methods**: This study included 2377 Mexican Mestizos (1378 ACS patients and 999 control individuals). The *PLTP* SNPs (rs6065904 *A/G*, and rs378114 *T/C*) were genotyped using TaqMan assays on Real-Time PCR equipment, following the manufacturer’s cycling protocol. **Results:** Logistic regression analysis under different inheritance models revealed that homozygosity for the rs6065904 *AA* and rs378114 *TT* genotypes was associated with a significant increased risk of ACS. In subgroup analysis restricted to ACS patients, both genotypes were also significantly associated with altered triglyceride and glucose levels. Complementary bioinformatic analysis using the Genotype-Tissue Expression (GTEx) Project suggested that these genotype–phenotype associations might be mechanistically linked to reduced *PLTP* mRNA expression. **Conclusions:** In summary, the rs6065904 *A/G* and rs378114 *T/C* SNPs in the *PLTP* gene are associated with ACS risk, and with circulating triglyceride and glucose levels.

## 1. Introduction

In recent decades, it has been established that genetic background, combined with cardiovascular risk factors such as lifestyle, dyslipidemia, obesity, elevated blood pressure, type 2 diabetes mellitus (T2DM), sex, age, and smoking, plays a major role in the early development of atherosclerotic plaques, which can lead to acute coronary syndrome (ACS) [1,2,3]. Among these risk factors, dyslipidemias represent primary drivers in the development of cardiovascular diseases, including ACS [1,2,3]. Recent evidence indicates that the *PLTP* gene, which encodes phospholipid transfer protein (PLTP), plays an important role in lipid and lipoprotein metabolism [4,5,6,7]. PLTP participates in high-density lipoprotein (HDL) remodeling through multiple pathways, shaping plasma HDL size distribution and mediating lipid transfer from triglyceride-rich lipoproteins to HDL during lipolysis [6,7]. Under physiological conditions, PLTP contributes to the formation of low-density lipoproteins (LDLs) from very-low-density lipoprotein (VLDL) particles and promotes HDL maturation [4,5,6,7,8].

Genetic analyses have shown that two single nucleotide polymorphisms (SNPs) in the intronic region of the *PLTP* gene, located on chromosome 20p14 (rs6065904 *A/G*, and rs378114 *T/C*; https://www.ncbi.nlm.nih.gov/snp/ (accesed on 3 February 2023)), contribute to susceptibility to hypercholesterolemia [4], coronary artery disease (CAD) [9,10], T2DM [11], and alterations in fatty acids and the plasma lipid profile [12,13,14]. Based on this information, we hypothesized that *PLTP* gene polymorphisms influence the early development of ACS through the dysregulation of plasma lipid levels. Therefore, this study aimed to determine whether the rs6065904 *A/G*, and rs378114 *T/C* polymorphisms of the *PLTP* gene are associated with alterations in plasma lipid levels and the risk of developing ACS.

## 2. Materials and Methods

### 2.1. Population

The sample size was calculated for an unmatched case–control study, using an alpha of 0.05 and a statistical power of 80% (http://www.openepi.com/SampleSize/SSCC.html (accesed on 7 July 2025)). The required minimum sample size was 1200 individuals (600 ACS and 600 controls). In total, 2377 individuals were enrolled: 999 control individuals and 1378 patients diagnosed with ACS. ACS patients were recruited between July 2018 and November 2025 at the Instituto Nacional de Cardiología Ignacio Chávez. The diagnosis of ACS was made following European Society of Cardiology (ESC) guidelines [15,16]; clinical manifestations, electrocardiographic findings, creatine kinase-MB, and cardiac troponin were determined for diagnosis. Exclusion criteria for patients with ACS comprised heart failure, liver disease, thyroid dysfunction, cancer, infectious diseases, or autoimmune disorders. The comparison group included 999 healthy participants of the Genetics of Atherosclerosis Disease (GEA) Mexican study, without personal or family history of cardiovascular diseases (CVDs) or heart failure, recruited between June 2008 and January 2013 [17]. Exclusion criteria for controls comprised renal, hepatic, thyroid, or oncological conditions, based on clinical history and biochemical testing. Both groups were ethnically matched and classified as Mexican Mestizos (defined as individuals of mixed Indigenous Amerindian and European ancestry born in Mexico). The study was conducted in strict compliance with the ethical standards set forth in the Declaration of Helsinki. All participants provided written informed consent. The institutional Ethics and Research Committees approved the study protocol (No. 25-1519).

### 2.2. Laboratory Analyses

Plasma samples were analyzed in the clinical laboratory on the day of collection for both ACS patients and healthy controls; frozen samples were not used for routine chemical chemistry determinations. Lipid profiles and glucose levels were measured using commercial kits from Randox Laboratories-US Ltd. (Kearneysville, WV, USA). HDL cholesterol (HDL-C) concentrations were measured after selective precipitation of apo B-containing lipoproteins (SPINREACT, Naucalpan de Mexico, Mexico). LDL cholesterol (LDL-C) was calculated using Friedewald’s formula for samples with triglyceride levels below 400 mg/dL [18]. Dyslipidemia was defined as total cholesterol > 200 mg/dL, LDL-C > 130 mg/dL, HDL-C < 40 mg/dL, or triglycerides > 150 mg/dL [19]. Patients were classified as diabetic if their fasting glucose exceeded 125 mg/dL or if they were receiving anti-diabetic medication [20]. Hypertension was defined as systolic blood pressure > 130 mmHg, diastolic blood pressure > 80 mmHg, or current use of oral antihypertensive treatment [21].

### 2.3. Genetic Analysis

DNA was obtained from peripheral blood cells using the QIAamp DNA Blood Mini Kit (QIAGEN, Hilden, Germany) and preserved at −80 °C until analysis. To minimize the likelihood of genotyping mistakes, DNA integrity was assessed prior to determining the polymorphisms. The *PLTP* SNPs (rs6065904 *A/G*, and rs378114 *T/C*) were genotyped using TaqMan assays on Real-Time PCR equipment, following the manufacturer’s cycling protocol. Thermal cycling consisted of an initial activation step at 95 °C for 10 min, followed by 40 cycles of 94 °C for 15 s and annealing step at 60 °C for 1 min.

### 2.4. Statistical Analysis

Hardy–Weinberg equilibrium was assessed in both groups using the chi-squared test. Statistical analysis was performed with SPSS version 18.0 (SPSS, Chicago, IL, USA). Data distribution was assessed using the Shapiro–Francia test. Normally distributed continuous variables are reported as mean ± standard deviation (SD) and were compared using Student’s *t*-test. Non-normally distributed variables are presented as median [interquartile range] and were analyzed using the Mann–Whitney U test. Categorical variables were evaluated using Pearson’s chi-squared test or Fisher’s exact test. To evaluate the association between rs6065904 *A/G* and rs378114 *T/C* SNPs of the *PLTP* gene with the susceptibility to development ACS, we used the following inheritance models: additive (major allele homozygotes versus heterozygotes versus minor allele homozygotes), codominant (major allele homozygotes versus minor allele homozygotes), dominant (major allele homozygotes versus heterozygotes + minor allele homozygotes), over-dominant (heterozygotes versus major allele homozygotes + minor allele homozygotes), and recessive (major allele homozygotes + heterozygotes versus minor allele homozygotes) using logistic regression [22,23]. All models were evaluated using multivariable logistic regression adjusted for sex, age, BMI, hypertension, smoking status, and T2DM. *p*-values were adjusted using Bonferroni correction (*pC*). Results are reported as odds ratios (ORs) with 95% confidence intervals (CIs). *p*-values < 0.05 were considered statistically significant.

### 2.5. Association of PLTP SNPs with Plasma Lipid and Glucose Levels

To assess the relationship between the rs6065904 *A/G* and rs378114 *T/C* SNPs and biochemical phenotypes (plasma lipids and glucose), ACS patients were stratified by genotype. Potential statistical associations between genotypes and metabolic parameters (total cholesterol, HDL-C, LDL-C, triglycerides, and glucose) were determined using the Kruskal–Wallis test, followed by post hoc Mann–Whitney U tests. *p*-values were corrected by Bonferroni test (*pC*). Outliers were removed prior to analysis to prevent statistical skewness.

### 2.6. Expression Quantitative Trait Locus (eQTL) Analysis

Quantitative trait locus (eQTL) analysis was performed using data from the Genotype-Tissue Expression (GTEx) Project (https://gtexportal.org/home/ (accesed on 15 September 2025)). This public resource evaluates human gene expression regulation and its relationship to genetic variation across diverse tissues. The software evaluates effects on SNPs on *PLTP* mRNA expression in relevant human tissues, including visceral adipose tissue, subcutaneous adipose tissue, coronary artery, left ventricle, and blood [24].

## 3. Results

### 3.1. Characteristics of the Study Sample

Table 1 summarizes the demographic and clinical characteristics of the study groups. Significant baseline differences were observed between groups. The ACS group contained a significantly higher proportion of males and had a higher prevalence of hypertension and active smoking (*p* < 0.001). Fasting glucose, systolic blood pressure, and diastolic blood pressure were significantly higher in ACS patients than in controls (*p* < 0.001). Conversely, total cholesterol, LDL-C, and HDL-C levels were significantly lower in ACS patients (*p* < 0.001), a finding likely attributable to acute statin administration.

### 3.2. Association of PLTP SNPs with ACS

Genotypic distributions for both SNPs agreed with HWE expectations in both groups (*p* > 0.05). Association analysis for rs6065904 *A/G* revealed that the *AA* genotype significantly increased the risk of developing ACS under codominant (OR = 1.39, *pC* = 0.027), recessive (OR = 1.40, *pC* = 0.007), and additive (OR = 1.16, *pC* = 0.039) models (Table 2). For rs378114 *T/C*, individuals carrying the *TT* genotype exhibited an elevated risk of ACS under codominant (OR = 4.37, *pC* = 0.023), recessive (OR = 4.17, *pC* = 0.013), and additive (OR = 1.39, *pC* = 0.028) models (Table 2).

### 3.3. Relationship of PLTP SNPs with Plasma Lipid Levels

**Figure 1:**
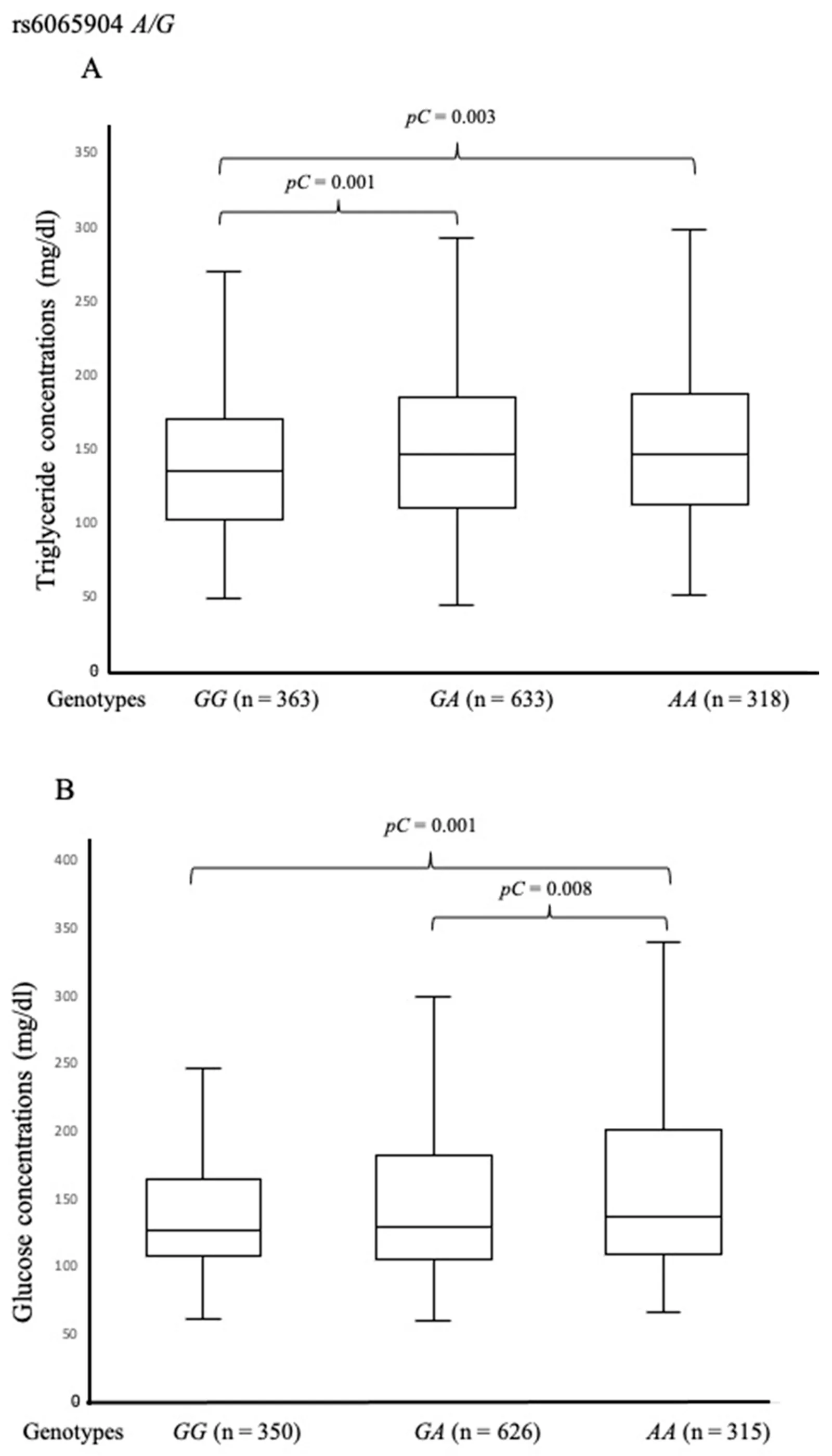
Plasma triglyceride (**A**) and glucose (**B**) concentrations in ACS patients stratified by *PLTP* rs6065904 *A/G* genotypes. Data are presented as median [interquartile range]. Intergroup differences were assessed using the Kruskal–Wallis test, followed by post hoc Mann–Whitney U tests. *p*-values were corrected by Bonferroni test (*pC*).

**Figure 2:**
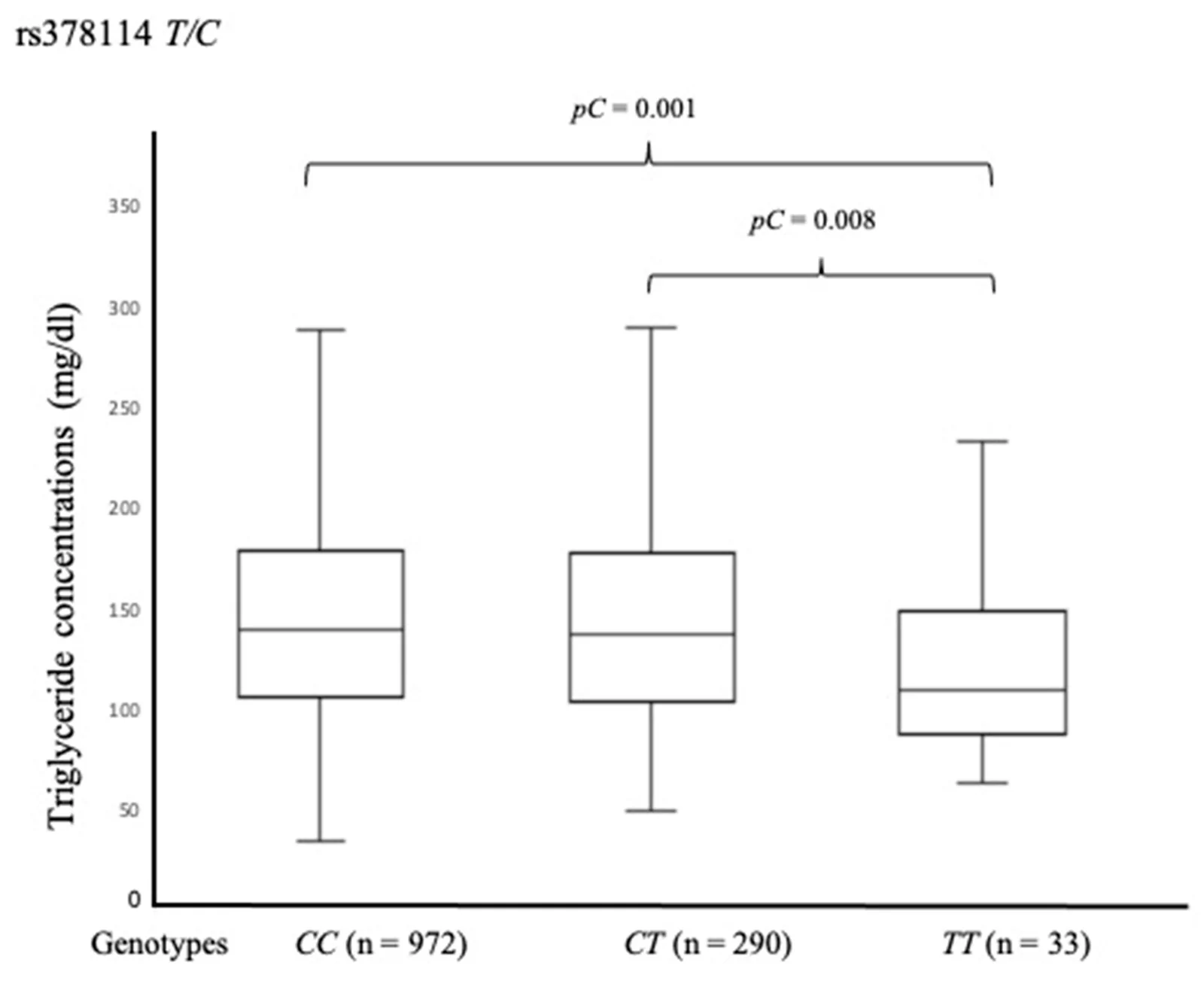
Plasma triglyceride concentrations in ACS patients stratified by *PLTP* rs378114 *T/C* genotypes. Data are presented as median [interquartile range]. Intergroup differences were assessed using the Kruskal–Wallis test, followed by post hoc Mann–Whitney U tests. *p*-values were corrected by Bonferroni test (*pC*).

Genotypic stratification in ACS patients demonstrated significant associations between *PLTP* SNPs and metabolic parameters. Carriers of the rs6065904 *GA* and *AA* genotypes displayed significantly higher triglyceride concentrations than *GG* homozygotes [146 mg/dL (110–185), *pC* = 0.001 and 146 mg/dL (112–187), *pC* = 0.003 vs. 135 mg/dL (103–169.5), respectively] (Figure 1A). Furthermore, the *AA* genotype was associated with higher glucose levels compared to *GA* and *GG* genotypes [137 mg/dL (109–201.5) mg/dL vs. 130 mg/dL (105–183) mg/dL, *pC* = 0.008; and vs. 128 (108–165) mg/dL, *pC* = 0.001, respectively] (Figure 1B). In contrast, analysis of rs378114 *T/C* showed that *TT* homozygotes had significantly lower triglyceride levels [110 mg/dL (88–150)] compared to *CT* [138 mg/dL (104–179), *pC* = 0.008] and *CC* carriers [140 mg/dL (107–180), *pC* = 0.001] (Figure 2).

### 3.4. Effect of PLTP Polymorphisms on eQTL Analysis

GTEx eQTL analysis confirmed that rs6065904 *A/G* and rs378114 *T/C* modulate *PLTP* mRNA expression. The rs6065904 *AA* genotype was associated with reduced *PLTP* mRNA expression in visceral adipose tissue, subcutaneous adipose tissue, and coronary artery tissue (*p* < 0.001) relative to the *GG* genotype (Supplementary Figure S1). Conversely, the rs378114 *TT* genotype was associated with increased *PLTP* mRNA expression across the same tissues (*p* < 0.001) relative to *CC* (Supplementary Figure S2).

## 4. Discussion

Plasma lipid alterations are central to the pathogenesis of cardiovascular diseases, including ACS. Over the past decade, studies investigating *PLTP* gene SNPs (rs6065904 *A/G* and rs378114 *T/C*) have yielded mixed findings regarding their impact on lipid profiles, cardiometabolic risk, and T2DM [9,10,11,25,26,27,28]. Our results demonstrate that the minor alleles (rs6065904 *A* and rs378114 *T*) are associated with an increased risk of ACS. Specifically, rs6065904 *AA* and *GA* genotypes correlated with higher plasma glucose and triglyceride levels, as well as reduced *PLTP* mRNA expression in adipose and coronary tissues, according to GTEx data [24]. Paradoxically, while the rs378114 *TT* genotype conferred an increased risk of ACS, *TT* homozygous patients exhibited lower triglyceride levels alongside higher tissue *PLTP* mRNA expression [24]. This suggests that the contribution of *PLTP* to ACS risk is not strictly mediated by overt dyslipidemia, as initially hypothesized in this study. Instead, PLTP participates in complex lipoprotein interactions, mediating the transfer of cholesterol and phospholipids from chylomicrons and VLDL to HDL, thereby altering particle size distribution and turnover [29,30,31]. Therefore, rather than simple contribution to plasma lipid levels, PLTP participates in the complex dynamic interactions between lipoproteins, which depends on the quantity and quality of donor and receptor particles [31,32]. To bridge the gap between the observed PLTP gene polymorphisms associations in this study and biological mechanisms, future functional studies are warranted. Given that PLTP plays a pivotal role in phospholipid transfer between lipoproteins [29,30,31], lipid delivery to cells may be altered and membrane lipid raft assembly impaired [33]. Then, macrophage and endothelial cell models could help to elucidate whether the rs6065904 and rs378114 variants alter membrane microdomain structure. Such structural changes could modify inflammation or nitric oxide synthetase signaling pathways [33], accelerating vascular inflammation in acute coronary events.

Moreover, some experimental evidence has demonstrated that *A* allele of the rs6065904 SNP is associated with low PLTP activity and low mRNA expression [11,26], findings that are in agreement with the GTEx data [24]. Beyond lipid transport, PLTP possesses anti-inflammatory properties through lipopolysaccharide (LPS) neutralization and maintains tissue vitamin E homeostasis [34,35]. Consequently, based on the results of this study and the broad ability of PLTP to interact with hydrophobic molecules, genetic variants of *PLTP* may serve as valuable markers of cardiovascular risk, independently of measurable lipid activity, which remains difficult to interpret. In this context, previous population studies report divergent outcomes. Jarvik et al. observed that the rs6065904 *A* and rs378114 *T* alleles were protective against carotid artery disease in Caucasian populations [25]. Vergeer et al. determined that the *C* allele of rs378114 and the *A* allele of rs6065904 were associated with low PLTP activity, and the combination of both polymorphisms determined liver mRNA expression, lower HDL size, and lower risk of cardiovascular disease in Caucasian populations [26]. Conversely, Zhao et al. reported the association of the rs6065904 *A* allele with increased CAD risk and elevated small HDL particles [9]. Genome-wide association and meta-analysis studies also link the rs6065904 and rs378114 *PLTP* SNPs with altered lipid profiles [12,13,14] and risk of cardiovascular diseases [10,27,28]. These discrepancies highlight the influence of ethnic variations in allele frequencies. In this context, the frequency of the *A* rs6065904 allele is substantially higher in Mexican Mestizos (44.7%) and Mexican-Americans in Los Angeles (50.0%) than in Caucasian (24.4%), Asian (33.7%), and African (21.2%) populations [36]. Conversely, the rs378114 *T* allele frequency is lower in Mexican Mestizos (11.7%) and Mexican-Americans (15.6%) compared to Caucasian (24.4%), Asian (38.3%), and African (42.9%) cohorts. Evaluating these SNPs in ethnically diverse populations is therefore necessary to establish their global role in atherogenesis and ACS [37] (Supplementary Table S1).

We acknowledge that our study has some limitations. First, samples from ACS patients and controls were independently collected in different periods that may introduce temporal bias; laboratory quality controls and DNA integrity analysis helped reduce this temporal factor, but it cannot be presumed that it was completely excluded. Second, the gender distribution was different between groups. However, male gender was considered in the statistical analyses as a covariable to statistically compensate this disparity. Also, *PLTP* polymorphisms associations with lipid levels have some limitations; lower levels of cholesterol in ACS patients than in controls clearly indicate an effect of the ACS-associated acute state and the use of statins as stated before. For this reason, the analysis of the PLTP gene polymorphisms on plasma lipid levels was performed only in ACS patients, assuming that the effect of statins and acute state is homogeneous in all the included individuals, but such an assumption cannot be verified. Finally, this study only showed a statistical relationship between genetic background and cardiovascular risk of developing ACS but did not establish the molecular mechanisms connecting *PLTP* variants to lipid and glucose shifts and ACS risk, which remain to be elucidated. Therefore, future experimental studies on mRNA expression such as luciferase assays, RT-qPCR (quantitative reverse transcription-polymerase chain reaction), or RNA sequencing (RNA-Seq) are required to ensure the validity and reliability of these polymorphisms in clinical practice.

## 5. Conclusions

Our findings showed that minor allele frequencies of the rs6065904 *A/G* and rs378114 *T/C* polymorphisms of the *PLTP* gene are associated with susceptibility to developing ACS and with triglyceride and glucose levels in the Mexican population. Further prospective studies across diverse ethnic groups are recommended to validate these variants as clinical biomarkers for cardiovascular risk.

## Figures and Tables

**Table 1:** Clinical and demographic parameters of the study groups.

Characteristics		ACS Patients (n = 1378)	Healthy Controls (n = 999)	*p*-Value
Age (years)		59 [52–67]	51 [45–57]	<0.001
BMI (kg/m^2^)		27.1 [24–29]	28 [25–31]	<0.001
Gender,	Male n (%)/Female n (%)	1108 (80.4)/270 (19.6)	404 (40.4)/595 (59.5)	<0.001
Hypertension,	Yes n (%)/No n (%)	796 (58)/582 (42)	229 (22.9)/770 (77.1)	<0.001
Smoking,	Yes n (%)/No n (%)	671 (49)/707 (51)	221 (22.1)/778 (77.9)	<0.001
T2DM,	Yes n (%)/No n (%)	792 (57.5)/586 (42.5)	---------	NS
Glucose (mg/dL)		136 [109–198]	89 [85–95]	<0.001
Systolic blood pressure (mmHg)		130 [115–149]	112 [103–122]	<0.001
Diastolic blood pressure (mmHg)		80 [70–90]	70 [65–76]	<0.001
Total cholesterol (mg/dL)		159 [127–193]	190 [167–211]	<0.001
HDL-C (mg/dL)		37 [31–44]	45 [36–55]	<0.001
LDL-C (mg/dL)		99 [72–129]	116 [95–134]	<0.001
Triglycerides (mg/dL)		143 [108–189]	143 [107–200]	NS

Continuous variables are presented as median [interquartile range] and analyzed using the Mann–Whitney U test. Categorical variables are shown as absolute frequencies and percentages. ACS = acute coronary syndrome; BMI = body mass index; T2DM = type 2 diabetes mellitus; HDL-C = high-density lipoprotein cholesterol; LDL-C = low-density lipoprotein cholesterol; NS = not significant.

**Table 2:** Association of PLTP gene polymorphisms with ACS according to inheritance models.

SNP (rsID-Number)/* Inheritance Model	Genotype	ACS Patients n = 1378 (n (%))	Controls n = 999 (n (%))	OR (95%CI)	*pC*
rs6065904 *A/G*					
Co-dominant	*GG*	382 (27.7)	300 (30.0)		
	*AG*	665 (48.3)	504 (50.5)		
	*AA*	331 (24.0)	195 (19.5)	1.39 (1.04–1.85)	0.027
Dominant	*GG*	382 (27.7)	300 (30.0)		
	*AG* + *AA*	996 (72.3)	699 (70.0)	1.09 (0.88–1.37)	0.431
Recessive	*GG* + *AG*	1047 (76.0)	804 (80.5)		
	*AA*	331 (24.0)	195 (19.5)	1.40 (1.09–1.79)	0.007
Heterozygous	*GG* + *AA*	713 (51.7)	495 (49.5)		
	*AG*	665 (48.3)	504 (50.5)	0.85 (0.70–1.05)	0.131
Additive	*--------*	---------	-----------	1.16 (1.01–1.34)	0.039
rs378114 *T/C*					
Co-dominant	*CC*	1038 (75.3)	775 (77.6)		
	*CT*	304 (22.1)	213 (21.3)		
	*TT*	36 (2.6)	11 (1.1)	4.37 (1.41–13.5)	0.023
Dominant	*CC*	1038 (75.3)	775 (77.6)		
	*CT* + *TT*	340 (24.7)	224 (22.4)	1.33 (0.96–1.84)	0.092
Recessive	*CC* + *CT*	1342 (97.4)	988 (98.9)		
	*TT*	36 (2.6)	11 (1.1)	4.17 (1.35–12.9)	0.013
Heterozygous	*CC* + *TT*	1074 (77.9)	786 (78.7)		
	*CT*	304 (22.1)	213 (21.3)	1.19 (0.85–1.66)	0.320
Additive	*--------*	---------	-----------	1.39 (1.04–1.88)	0.028

SNP = single nucleotide polymorphism; ACS = acute coronary syndrome; OR = odds ratio; CI = confidence interval; *pC* = Bonferroni-corrected *p*-value. * Inheritance models were designed based on the minor allele. All models were evaluated using multivariable logistic regression adjusted for sex, age, BMI, hypertension, smoking status, and T2DM.

## Data Availability

The data presented in this study are available from the corresponding author upon request, due to privacy, legal, or ethical reasons.

## Supplementary Materials

The following supporting information can be downloaded at: https://www.mdpi.com/article/10.3390/biomedicines14092025/s1, Supplementary Table S1. Allele frequencies of PLTP polymorphisms across reference populations. Supplementary Figure S1. According with the expression quantitative trait locus (eQTL) analysis of Genotype-Tissue Expression (GTEx) consortium data, the rs6065904 *AA* genotype leads to lower mRNA expression in the visceral adipose tissue (A), subcutaneous adipose tissue (B), and artery coronary (C). Supplementary Figure S2. According with the expression quantitative trait locus (eQTL) analysis of Genotype-Tissue Expression (GTEx), the rs378114 *TT* genotype showed a higher mRNA expression in the visceral adipose tissue (A), subcutaneous adipose tissue (B), and artery coronary (C).
