# Supplementary material for: PLTP Variants rs6065904 and rs378114 Are Associated with Acute Coronary Syndrome Risk and Altered Glucose and Triglyceride Levels in a Mexican Population
Source: Biomedicines. 2026 Sep 9;14(9):2025. doi: 10.3390/biomedicines14092025 (PMC13604363; doi:10.3390/biomedicines14092025)
Supplement: Supplementary file 1 [file biomedicines-14-02025-s001.zip › biomedicines-4513105-supplementary.pdf]

Supplementary Table S1. Allele frequencies of PLTP polymorphisms across reference populations.

SNP/Alleles	Mexican Mestizos		Mexican with Ancestry in Los Angeles.		Caucasian*		Asian*		African*		References
rs6065904 *A/G*											
	(n=999)		(n=64)		(n=503)		(n=504)		(n=661)		
Allele	n	af	n	af	n	af	n	af	n	af	
G	1104	55.2	64	50.0	761	75.6	668	66.3	1042	78.8	Present study[A]
A	894	44.7[A]	64	50.0	245	24.4	340	33.7	280	21.2	
rs378114 *T/C*											
	(n=999)		(n=64)		(n=503)		(n=504)		(n=661)		
Allele	n	af	n	af	n	af	n	af	n	af	
C	1763	88.2	108	84.4	761	75.6	622	61.7	755	57.9	Present study[B]
T	235	11.7[B]	20	15.6	245	24.4	386	38.3	567	42.9	

SNP = single nucleotide polymorphism; af = allele frequency. Data for reference populations were obtained of *Ensembl Platform: https://www.ensembl.org/Homo_sapiens/Variation/Population?db=core;r=20:45905512-45906512;v=rs6065904;vdb=variation;vf=1054672306, and https://www.ensembl.org/Homo_sapiens/Variation/Population?db=core;r=20:45909288-45910288;v=rs378114;vdb=variation;vf=1054554809 (accessed on 20 January 2026)).

[A] The rs6065904 A allele frequency was higher in Mexican Mestizos and Mexican-Americans in Los Angeles than in Caucasian, Asian, and African populations.

[B] The rs378114 T allele frequency in Mexican Mestizos and Mexican-Americans in Los Angeles was lower compared to Caucasian, Asian, and African populations.

rs6065904 *A/G*

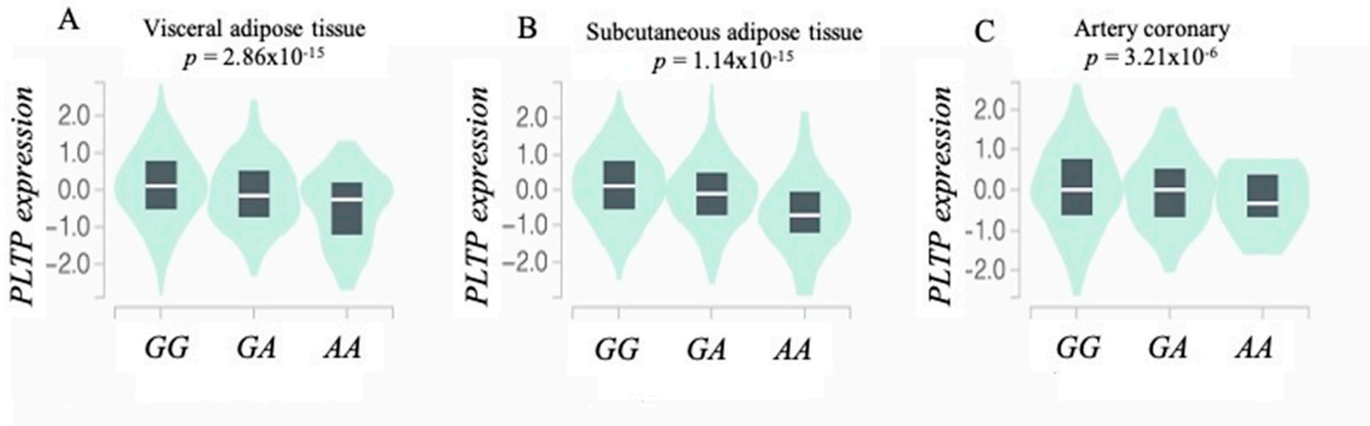

Supplementary Figure S1. According, with the expression quantitative trait locus (eQTL) analysis of Genotype-Tissue Expression (GTEx) consortium data, the rs6065904 *AA* genotype leads to lower mRNA expression in the visceral adipose tissue (**A**), subcutaneous adipose tissue (**B**), and artery coronary (**C**).

rs378114 *T/C*

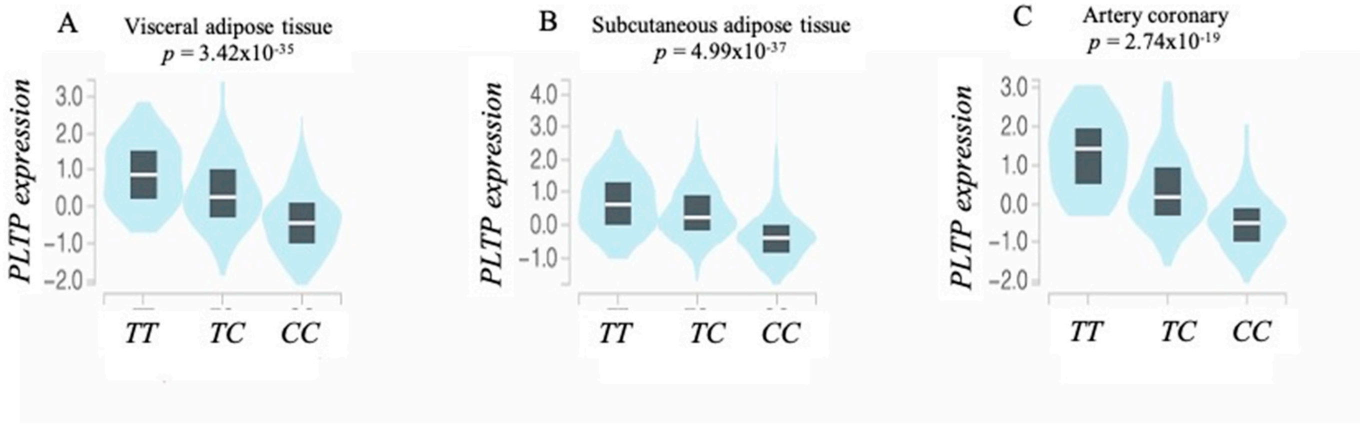

Supplementary Figure S2. According with the expression quantitative trait locus (eQTL) analysis of Genotype-Tissue Expression (GTEx), the rs378114 *TT* genotype showed a higher mRNA expression in the visceral adipose tissue (**A**), subcutaneous adipose tissue (**B**), and artery coronary (**C**).
